# Effects of Achievement Goals on Challenge Seeking and Feedback Processing: Behavioral and fMRI Evidence

**DOI:** 10.1371/journal.pone.0107254

**Published:** 2014-09-24

**Authors:** Woogul Lee, Sung-il Kim

**Affiliations:** Department of Education and *b*MRI (Brain and Motivation Research Institute), Korea University, Seoul, Korea; University of Western Ontario, Canada

## Abstract

We conducted behavioral and functional magnetic resonance imaging (fMRI) research to investigate the effects of two types of achievement goals—mastery goals and performance-approach goals— on challenge seeking and feedback processing. The results of the behavioral experiment indicated that mastery goals were associated with a tendency to seek challenge, both before and after experiencing difficulty during task performance, whereas performance-approach goals were related to a tendency to avoid challenge after encountering difficulty during task performance. The fMRI experiment uncovered a significant decrease in ventral striatal activity when participants received negative feedback for any task type and both forms of achievement goals. During the processing of negative feedback for the rule-finding task, performance-approach-oriented participants showed a substantial reduction in activity in the dorsolateral prefrontal cortex (DLPFC) and the frontopolar cortex, whereas mastery-oriented participants showed little change. These results suggest that performance-approach-oriented participants are less likely to either recruit control processes in response to negative feedback or focus on task-relevant information provided alongside the negative feedback. In contrast, mastery-oriented participants are more likely to modulate aversive valuations to negative feedback and focus on the constructive elements of feedback in order to attain their task goals. We conclude that performance-approach goals lead to a reluctant stance towards difficulty, while mastery goals encourage a proactive stance.

## Introduction

Achievement goal theory is a central explanatory framework in human motivation studies [1], [2]. Achievement goal theorists differentiate between two distinct types of achievement goals: mastery and performance goals [3]. Mastery-oriented individuals endeavor to improve their competence by gaining new knowledge or improving their skills, whereas performance-oriented individuals try to demonstrate their competence by outperforming others or gaining public recognition for their ability. These two groups tend to have different understandings of human ability, influencing the decision to seek or avoid challenge in the face of difficulty or obstacles [4]. Mastery-oriented individuals commonly hold the belief that human ability is malleable and that difficulty can be overcome through effort; as a result, they are more willing to overcome problems encountered in pursuit of their goals. However, performance-oriented individuals prefer to view human ability as fixed, and that difficulty cannot be easily overcome regardless of the level of effort expended. This attitude leads to a habit of avoiding difficulty to protect self-esteem. Based on this conceptual divide, achievement goal theorists generally agree that performance goals have maladaptive relations with learning and motivation, while mastery goals have positive influences [3], [4].

More recently, however, some researchers have insisted that possible benefits of performance goals have been ignored or minimized because performance goals are broadly conceptualized and, therefore, proposed a revision of the performance goals, dividing them into two types: adaptive (i.e., performance-approach goals) and maladaptive (i.e., performance-avoidance goals) types [5], [6]. This idea has been supported by the findings that performance-approach goals are positively, or at least non-negatively, related to beneficial learning processes and outcomes; performance-avoidance goals, on the other hand, are generally acknowledged to have detrimental effects [7], [8]. Until now, little evidence has been accumulated to determine whether performance-approach goals also have a positive effect on motivation in the face of difficulty, even though it has been firmly established performance-avoidance goals have a negative relation with challenge-seeking behavior after failure [9]. Therefore, we aimed to compare the two adaptive types of goals (i.e., mastery and performance-approach goals) in terms of challenge seeking and brain activation patterns during negative feedback processing.

In a behavioral experiment, we investigated the relation between achievement goals and the desire to seek challenge. Based on the previous findings [9]–[11], our first hypothesis was that mastery-oriented individuals would choose difficult tasks even after experiencing difficulty during task performance, in contrast to performance-approach-oriented individuals, who would choose easier tasks. This difference in challenge-seeking behavior may be due to contrasting perceptions of both task performance difficulty and negative performance feedback [4], [12]. Mastery-oriented individuals are likely to view difficulty as a natural part of learning and to perceive negative feedback as useful information for subsequent learning. Performance-approach-oriented individuals, however, are likely to view difficulty as a negative outcome of learning and to perceive negative feedback as threatening [9].

In order to investigate whether mastery-oriented and performance-approach-oriented individuals react differently to negative feedback, we also conducted a functional magnetic resonance imaging (fMRI) experiment. To differentiate the nature of performance feedback (i.e., more confirmatory vs. more informative), we used two different experimental tasks: multiplication and rule-finding tasks. Previous neuroscience studies have revealed that, regardless of feedback type, feedback valence modulates the neural activity related to reward processing such as ventral striatal activity [13]–[15]. Therefore, our second hypothesis was that there would be a decrease in the ventral striatal activity during negative feedback, whereas there would be an increase during positive feedback.

Although it is generally accepted that performance-approach-oriented individuals are more vulnerable to negative feedback than are mastery-oriented individuals [11], we expected that this would be more pronounced when the feedback was more informative. When negative feedback contains information useful for successful task performance (i.e., negative feedback provided in the rule-finding task), we hypothesized that mastery-oriented individuals would modulate aversive valuations to the negative feedback and utilize the task-relevant information for future performance, whereas performance-approach-oriented individuals would not. This third hypothesis is also supported by previous neuroscience findings that the neural activity related to aversive valuations can be decreased by the control of prefrontal activity, particularly the dorsolateral prefrontal cortex (DLPFC) [16]. Though the DLPFC is known to be associated with diverse cognitive processes [17], it is well established that the DLPFC is recruited when individuals need to recruit control processes in response to negative feedback during task performance [18], [19]. As mastery-oriented individuals tend to focus more on the task-relevant value of feedback, in contrast to performance-oriented individuals who tend to focus more on feedback valence [12], we hypothesized that mastery-oriented individuals would show greater neural activity related to cognitive control (e.g., DLPFC activity) while performance-approach-oriented individuals would not.

During feedback processing in the rule-finding task, higher-order executive functions can also be recruited differently depending on achievement goal type. Mastery-oriented individuals tend to maintain their concentration on a task following negative feedback perceiving it as informative as positive feedback for the attainment of a long-term task goal. Conversely, performance-approach-oriented individuals tend to lose focus in response to negative feedback seeing it as a sign of failure [4], [11]. Thus, our fourth hypothesis was that mastery-oriented individuals would show little change in neural activity related to higher-order executive functions such as frontopolar cortex activity [20], [21], when positive and negative feedback are compared, whereas performance-approach-oriented individuals would show a substantial reduction in frontopolar cortex activity during negative feedback when compared to positive feedback.

## Method

### Participants of the behavioral experiment

For the behavioral experiment, we recruited 161 Korean undergraduate students (69 males and 92 females) from introductory psychology classes at a university in Korea. We excluded 11 participants who either did not respond to the survey or did not perform the experimental task. Thus, the data of 150 participants (65 males and 85 females) were included in the final analyses.

### Measures of the behavioral experiment

Mastery goals and performance-approach goals were measured using the Korean version, developed by Park and Lee [22], of an achievement goal questionnaire based on a 2×2 achievement goal framework [23]. We used ten items with a five-point Likert scale to assess mastery goals (e.g., “It is important for me to understand the content of this task as thoroughly as possible.”; α = .75) and ten items with a five-point Likert scale to assess performance-approach goals (e.g., “It is important for me to do well compared to others in this task.”; α = .89).

In the case of math self-efficacy, 10 items were adopted from the Korean version of a math self-efficacy questionnaire developed by So and Kim [24]. Example items were “Compared with others, I am very good at math.” and “I am good at solving math problems.” (α = .77).

To assess challenge-seeking behavior prior to performing the experimental task, we adopted four items (e.g., “I prefer solving difficult math problems to solving easy ones.”; α = .74) with a five-point Likert scale from previous achievement goal research [10], [11].

### Task and procedure of the behavioral experiment

The behavioral experiment was conducted with three different classes. In each class, 50–60 participants each completed a questionnaire regarding their achievement goals, math self-efficacy, and challenge seeking before performing the experimental task. After receiving instructions, participants were asked to complete the task within five minutes. The task was to solve a series of numerical progression problems that alternated between easy and difficult. Each problem contained a blank, and the participants were asked to identify the correct number to complete the progression. The easy problem set, named “Type A” in the experiment, consisted of four easy problems (e.g., 2, 4, 8, 16, [ ], 64), whereas the difficult problem set, named “Type B”, consisted of two moderately difficult problems (e.g., 20, 38, [ ], 68, 80, 90) and two extremely difficult problems (e.g., 1, 3, 5, 7, [ ], 131). The extremely difficult problems were manipulated to ensure that the participants experienced difficulty finding the correct answers, leading them to eventually fail to solve the problems within the set time limit.

After the participants completed the task, the experimenter asked them to exchange their answer sheets with the people next to them. Then, the correct answers for the problems were provided, and the participants were asked to mark the score for each of the easy and difficult problem sets on the answer sheets (e.g., three correct answers out of four “Type A” problems). After the participants got their answer sheets back, they were asked to decide which of the easy and difficult problem sets they wanted to perform in a follow-up task. This decision was used as an indicator of their willingness to seek challenge after experiencing difficulty during task performance. There was, however, no actual follow-up task given, and the experiment ended once the participants had made their choice.

### Participants of the fMRI experiment

Participants for the fMRI study were recruited from the 150 participants of the behavioral experiment; we selected 13 mastery-oriented participants (6 females and 7 males; between the ages of 18 and 28) and 14 performance-approach-oriented participants (7 females and 7 males; between the ages of 18 and 27). Mastery-oriented participants showed marginally stronger self-reported mastery goals (*M* = 3.52, *SD* = .37) than performance-approach-oriented participants did (*M* = 3.14, *SD* = .60; *F*(1,25) = 3.90, *p* = .059). In addition, mastery-oriented participants chose the difficult task in the behavioral experiment. In contrast, performance-approach-oriented participants showed stronger self-reported performance-approach goals (*M* = 3.76, *SD* = .45) than mastery-oriented participants did (*M* = 2.59, *SD* = .67; *F*(1,25) = 28.71, *p*<.05) and chose the easy task in the behavioral experiment. The participants were selected so each group had a similar distribution of performance scores for both tasks in the behavioral experiment. For the easy task, the mean performance score was 3.77±.44 out of four problems for mastery-oriented participants and 3.71±.47 for performance-approach-oriented participants (*F*(1,25) = .10, *p*>.05), and for the difficult task, the mean performance score was 1.46±.52 out of four problems for mastery-oriented participants and 1.43±.51 for performance-approach-oriented participants (*F*(1,25) = .03, *p*>.05). Finally, participants were also chosen to establish similar perceived self-efficacy (*M* = 2.83±.55 for mastery-oriented participants; *M* = 2.94±.54 for performance-approach-oriented participants; *F*(1,25) = .25, *p*>.05). All participants were right-handed and had no history of neurological illness. They voluntarily signed an informed consent form and received approximately $30 for their participation. This study was approved by the Institutional Review Board of Korea University. The data for one female performance-approach-oriented participant were excluded because of excessive head movement during fMRI scanning (greater than 2.0 mm in any directions). Thus, the data from 13 mastery-oriented and 13 performance-approach-oriented participants were included in the subsequent analyses.

### Task and procedure of the fMRI experiment

The fMRI experiment was an event-related experiment consisting of four runs: two runs in which participants were presented with a multiplication task and other two runs in which they were presented with a rule-finding task. These four runs were alternated between the multiplication and rule-finding tasks. Each run, which began with a 10-second instruction page, consisted of 30 trials and lasted 370 seconds.

**Figure 1:**
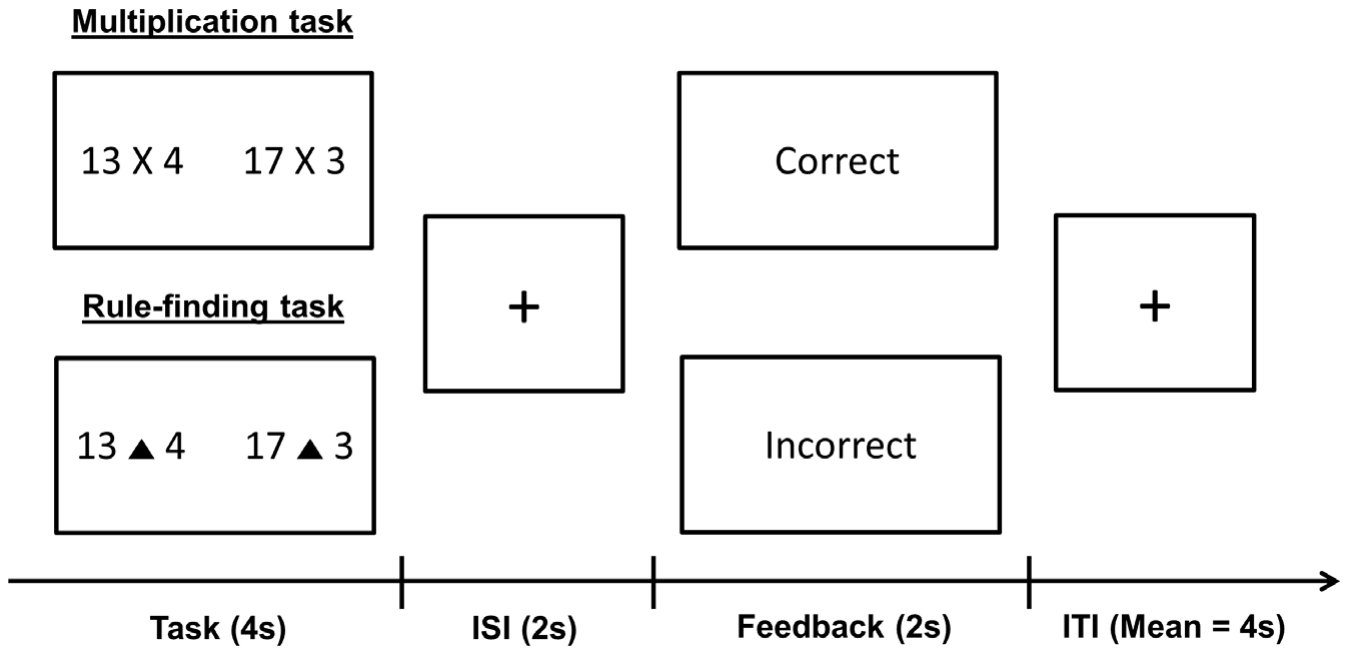
Procedure for each trial: The instruction page was presented for ten seconds at the beginning of each run. During the four-second presentation of the multiplication or rule-finding problem, the participants were required to decide which of the two results was greater. After a two-second inter-stimulus interval (ISI), the participants received a 2-second positive or negative feedback stimulus contingent upon their answer. The subsequent trial was presented after a randomized inter-trial interval (ITI) lasting 1–15 seconds (M = 4 seconds).

In each trial of the multiplication task (Figure 1), the participants perform a multiplication problem, randomly selected from the 30 different problems. The multiplication problems were required to determine which of two multiplication results was greater (e.g., 13×4 vs. 17×3). Participants were required to calculate two multiplication problems and compare the results within a set time limit. This time limit, which was determined based on the results of pilot tests, made the task optimally challenging. The participants were asked to press the left button if they thought that the multiplication result on the left was greater and the right button if they thought that the one on the right was greater. After a two-second inter-stimulus interval, the participants received two seconds of positive feedback (i.e., correct) or negative feedback (i.e., incorrect). The participants received negative feedback if they did not respond within the time limit. In this task, feedback only relayed the accuracy of the answer to the participants, and so did not contain any task-relevant information that participants could use for subsequent performance. The next trial was presented after a randomized inter-trial interval lasting between 1–15 seconds (*M* = 4 seconds), calculated using OptSeq (http://surfer.nmr.mgh.harvard.edu/optseq/). Therefore, the mean duration for each trial was 12 seconds.

In each trial of the rule-finding task (Figure 1), the participants were required to determine which of two results produced by an unknown arithmetic operation was greater within four seconds. The sequence for each trial (i.e., the performance phase, the two-second ISI, the two seconds of feedback contingent upon the participant's answer) and the numbers used for each problem (e.g., 13 ▴ 4 vs. 17 ▴ 3) were identical to those used in the multiplication task. The only difference was that the multiplication sign (i.e., x) was substituted for an artificial arithmetic operator (e.g., ▴) in the rule-finding task. The same artificial arithmetic operator, which was supposed to represent an unknown arithmetic rule (e.g., “A ▴ B” means to choose a bigger number between the value of A and twice the value of B.), was presented throughout each run in the rule-finding task. Participants were asked to calculate two arithmetic problems according to their supposed arithmetic rule and compare the results within a time limit. Ultimately, participants were required to determine the unknown arithmetic rule by performing continuous trials. Thus, the performance feedback in this task informed participants whether their working hypothesis regarding the arithmetic rule was correct or not, which was crucial for guiding subsequent performance. Although a valid rule was used, it was extremely difficult to determine, forcing participants to continuously generate alternative rules. Post-experiment questions indicated that all participants reported their own supposed arithmetic rule for each unknown arithmetic sign but failed to find the correct one.

Participants received instructions on the task and became familiar with the process of determining the unknown arithmetic rule through practice trials before scanning commenced. Functional images were acquired while they performed the task, and anatomical brain images were acquired at the end of the brain scan. In the post-experiment questions, the experimenter asked what participants' supposed arithmetic rule for each rule-finding task run was and checked whether it was correct. At the end of the experiment, the experimenter debriefed about the experiment.

### fMRI data acquisition

A 3T FORTE scanner (ISOL Technology, Korea) was used for acquiring anatomic and functional images. Using a T2*-weighted single shot gradient echo planar imaging (EPI) sequence sensitive to blood oxygenation level-dependent (BOLD) contrast (TR = 2000 ms, TE = 35 ms, flip angle  = 60°, field of view  = 240 mm, 64×64 matrix, in-plane resolution  = 3.75×3.75, and slice thickness  = 5 mm with no gap), 25-slice functional images were acquired. Then, high-resolution T1-weighted anatomic images were obtained to precisely determine the structures corresponding to the functional activation foci (TR = 10 ms, TE = 5.7 ms, flip angle  = 10°, and voxel size  = 1×1×1 mm^3^).

### fMRI data analysis

AFNI (http://afni.nimh.nih.gov) [25] was used to preprocess the images and to conduct the statistical analyses. To exclude the effects of unstable hemodynamics and MRI signals, the first five images of each run were discarded. In preprocessing, the functional images were spatially and temporally realigned for data correction. The realigned data were spatially blurred with a 5 mm Gaussian kernel of which full-width at half-maximum (FWHM) and scaled for subsequent analyses.

In individual analyses, the preprocessed data were analyzed using a general linear model (GLM) with ten regressors, with analyses performed separately for the multiplication and rule-finding tasks. We concluded that neural activity between the multiplication and rule-finding tasks could not be compared because the trials for the multiplication and rule-finding tasks were presented in separate runs and, as a result, separately by a significant period of time. We had recognized this limitation in the design of the study but nevertheless did not shuffle the trials for these two tasks because the trials for each rule-finding run needed to be presented together under the same unknown arithmetic rule. Two of the regressors in the model represented the experimental condition: positive feedback and negative feedback. To control for the confounding effects of task performance and head motion, we included two regressors for task performance (i.e., the time points of participants' responses to the problems) of the positive and negative feedback conditions and six regressors for head motion parameters. The beta weights of the regressors for each experimental condition were used for group analyses. For the group analyses, the individual statistical data were matched to the Montreal Neurological Institute (MNI) template and resampled to 2×2×2 mm^3^ voxels.

In the group analyses, subtraction analyses between positive and negative feedback were conducted separately for each experimental task to compare neural activity during feedback processing for mastery-oriented and performance-approach-oriented participants. To correct the multiple comparison problem in the subtraction analyses, we considered both voxel-wise threshold (*p*<.005) and cluster size (*n*≥56, a minimum volume of 448 mm^3^), which set a cluster-wise corrected *p* value of.046 [26]. We examined BOLD signal changes in regions of interests (ROI) activated in the subtraction analyses between positive and negative feedback in order to compare the neural activation patterns between experimental conditions. In order to avoid the issue of non-independence bias [27], we utilized the leave-one-subject-out (LOSO) method [28], thereby making the subtraction and ROI analyses independent. In the LOSO method, we omitted GLM data for one participant and ran independent group analyses by only making use of the remaining participants' data to define independent clusters of the ROI. Each ROI was sphere 5 mm in diameter centered on the peak coordinates of the activation. We then extracted the BOLD signals in the ROI from the left-out participants' data.

Psychophysiological interaction (PPI) analyses [29] were also carried out separately for each experimental task to examine the possibility that the brain regions related to the control process (e.g., dorsolateral prefrontal cortex activity) interact differently with reward-related neural activity according to feedback valence and achievement goal orientation. The seed brain regions for the PPI analyses were the ventral striatum activated in the subtraction analysis between positive and negative feedback for each experimental task. To correct the multiple comparison problem in the PPI analyses, we also used the cluster-wise threshold (*p*<.048), which was determined by both voxel-wise threshold (*p*<.005) and cluster size (*n*≥53, a minimum volume of 424 mm^3^). We used Talairach coordinates [30], converted from the MNI coordinates, to report the brain regions activated in the subtraction and PPI analyses.

## Results

### Results of the behavioral experiment

We conducted a paired *t*-test to determine whether the manipulation of the easy and difficult tasks worked as planned. Participants performed much better on the easy task (*M* = 3.38, *SD* = .92) than on the difficult task (*M* = 1.23, *SD* = .67; *t*(149) = 29.70, *p*<.05), indicating that the manipulation of task difficulty was successful.

The descriptive statistics and intercorrelations among the seven measures assessed in the behavioral experiment appear in Table 1. Table 2 shows how the achievement goals and statistical controls (i.e., task performance, math self-efficacy) predicted the pre-task and post-task challenge seeking outcomes using a hierarchical regression analysis for pre-task challenge seeking and a hierarchical logistic regression analysis for post-task challenge seeking. For each analysis, the variables for perceived present ability (math self-efficacy for pre-task challenge seeking; math self-efficacy and performance in the easy and difficult tasks for post-task challenge seeking) were ordered first as covariates; mastery goals and performance-approach goals were simultaneously added in the second block; and the interaction term for mastery and performance-approach goals was added in the last block.

**Table 1:** Descriptive and Correlational Statistics.

Variable	1	2	3	4	5	6	7
1. Mastery goals	—						
2. Performance-approach goals	.11	—					
3. Easy task performance	−.05	.09	—				
4. Difficult task performance	−.03	.13	.41**	—			
5. Math self-efficacy	.29**	.11	.27**	.17*	—		
6. Pre-task challenge seeking	.36**	.10	.24**	.21**	.69**	—	
7. Post-task challenge seeking	.23**	−.09	.13	.17*	.17*	.27**	—
Possible range	1–5	1–5	0–4	0–4	1–5	1–5	0 or 1
Mean	3.32	2.99	3.38	1.23	2.86	2.78	.23
Standard deviation	.48	.70	.92	.67	.56	.82	.42
Skewness	−.11	−.05	−1.61	−.16	.01	.21	1.27
Kurtosis	.06	.31	2.17	−.54	.31	−.36	−.38

*Note*. Post-task challenge seeking was coded 1 when participants chose the difficult task and 0 when they chose the easy task as a follow-up task.

*p<.05,

**p<.01. *N* = 150.

**Table 2:** Standardized Beta Coefficients of Variables.

Explanatory variable	Pre-task challenge seeking	Post-task challenge seeking
Math self-efficacy	.63**	.12
Easy task performance	—	.14
Difficult task performance	—	.23
Mastery goals	.18**	.35**
Performance-approach goals	.01	−.24*

*Note*. Beta coefficients were examined in the regression model with math self-efficacy, mastery goals, and performance-approach goals for pre-task challenge seeking and in the regression model with math self-efficacy, easy and difficult task performance, mastery goals, and performance-approach goals for post-task challenge seeking.

*p<.05,

**p<.01. *N* = 150.

After the effects of the covariates were partialled out, there was an additional unique contribution of achievement goals to explain the variance of challenge seeking (Δ*F*(2, 146) = 5.35, *p*<.05 for pre-task challenge seeking; Δ*χ* ^2^(2) = 10.82, *p*<.05 for post-task challenge seeking), but there was no such contribution by their interaction (Δ*F*(1, 145) = .88, *p*>.05 for pre-task challenge seeking; Δ*χ* ^2^(1) = .71, *p*>.05 for post-task challenge seeking). Examination of the beta coefficients in the regression models (Table 2) showed that performance-approach goals had null relations with pre-task challenge seeking (β = .01, *p*>.05), whereas mastery goals had positive relations (β = .18, *p*<.01). Interestingly, math self-efficacy was positively related to pre-task challenge seeking even when achievement goals were considered (β = .63, *p*<.01). For post-task challenge seeking, however, performance-approach goals demonstrated negative relations (β = −.24, *p*<.05), whereas mastery goals had positive relations (β = .35, *p*<.01).

### Results of the fMRI experiment

#### Behavioral results

There was no difference in mean accuracy rates between mastery-oriented and performance-approach-oriented participants for either the multiplication task (*M* = 63.1%, *SEM* = 3.60% for the mastery group and *M* = 63.3%, *SEM* = 2.75% for the performance-approach group; *F*(1,24) = .003, *p*>.05) or the rule-finding task (*M* = 54.6%, *SEM* = 3.95% for the mastery group and *M* = 51.9%, *SEM* = 3.69% for the performance-approach group; *F*(1,24) = .25, *p*>.05). This indicates that both participant groups received similar amounts of positive and negative feedback for each task type.

In the multiplication task, all participants had faster reaction times (RT) in the trials where they received positive feedback compared to those where they received negative feedback (*F*(1,24) = 9.27, *p*<.05). Except for this, however, there were no other significant differences in mean RT when comparing achievement goal orientation (*F*(1,24) = 1.89, *p*>.05) and its interaction with feedback type (*F*(1,24) = .99, *p*>.05). In the rule-finding task, no mean RT differences were observed depending on feedback type (*F*(1,24) = 3.30, *p*>.05), achievement goal orientation (*F*(1,24) = .72, *p*>.05), and their interaction (*F*(1,24) = .05, *p*>.05). For the same feedback type, there were no mean RT differences depending on the task type (*F*(1,24) = 3.46, *p*>.05 for positive feedback; *F*(1,24) = 4.11, *p*>.05 for negative feedback), achievement goal orientation (*F*(1,24) = .24, *p*>.05 for positive feedback; *F*(1,24) = .40, *p*>.05 for negative feedback), and their interaction (*F*(1,24) = 1.75, *p*>.05 for positive feedback; *F*(1,24) = .31, *p*>.05 for negative feedback) (Table 3). These results indicate that the participants spent an equal amount of time on the rule-finding task as they did on the multiplication task.

**Table 3:** Mean RT across Conditions.

	Multiplication task		Rule-finding task	
Group	Po	Ne	Po	Ne
MG	2881.4±54.8	2977.3±43.3	2843.2±74.9	2883.2±75.6
PG	3010.5±61.0	3059.2±65.7	2784.7±89.5	2894.5±84.6

*Note*. The mean RT for each condition is presented in milliseconds. MG: mastery-oriented participants, PG: performance-approach-oriented participants, Po: positive feedback, Ne: negative feedback.

#### fMRI results of the multiplication task

**Figure 2:**
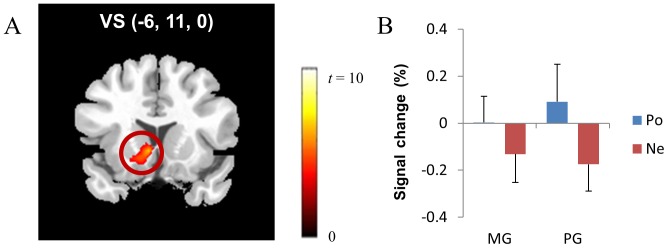
In the multiplication task, the ventral striatum (VS) (A) showed greater neural activity during positive feedback than during negative feedback (corrected *p*<.046). BOLD signal changes in the ventral striatum were examined (B). *Note*. MG: mastery-oriented participants, PG: performance-approach-oriented participants, Po: positive feedback, Ne: negative feedback.

The left ventral striatum (Figure 2A) and the left angular gyrus were more activated during positive feedback than negative feedback (corrected *p*<.046; Table 4), whereas no brain regions exhibited greater neural activity during negative feedback when compared to positive feedback. The greater neural activity in the ventral striatum during positive feedback was consistent with our hypothesized relations between ventral striatal activity and hedonic reactions.

**Table 4:** Results of the Subtraction Analysis between Positive and Negative Feedback in the Multiplication Task.

				Talairach Coordinates			
Region	BA	Volume	Side	x	y	z	Maximum *t* value
***Positive – Negative Feedback***							
Ventral striatum		3104	L	−6	11	0	7.20
Angular gyrus	39	968	L	−34	−59	34	4.28

*Note*. The cluster-wise threshold (correct *p*<.046) is determined by voxel-wise threshold (*p*<.005) and the minimum volume (56 contiguous voxels; 448 mm^3^).

To determine the effect of achievement goal type on the neural responses to positive and negative feedback, we examined BOLD signal changes in the left ventral striatum as determined by the subtraction analysis between positive and negative feedback. Although feedback valence had a significant effect on ventral striatal activity (*F*(1,24) = 13.68, *p*<.01), the effect of achievement goal type (*F*(1,24) = .02, *p*>.05) and the two-way interaction effect between feedback valence and achievement goal type (*F*(1,24) = 1.45, *p*>.05) were not significant (Figure 2B). In other words, participants with different types of achievement goals had similar brain activation patterns: increased ventral striatal activity in response to positive feedback and decreased ventral striatal activity in response to negative feedback.

**Figure 3:**
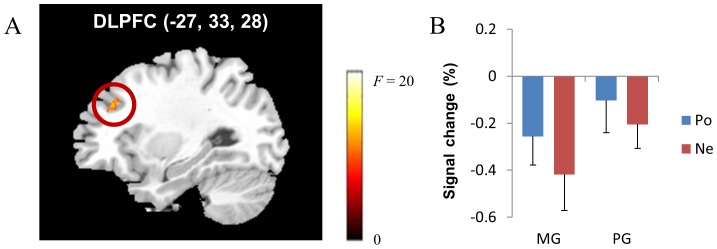
In the multiplication task, ventral striatal activity had a greater positive interaction with dorsolateral prefrontal cortex (DLPFC) activity (A) during negative feedback than during positive feedback regardless of achievement goal orientation (corrected *p*<.048). The pattern of BOLD signal changes in the DLPFC is presented (B). *Note*. MG: mastery-oriented participants, PG: performance-approach-oriented participants, Po: positive feedback, Ne: negative feedback.

We conducted a PPI analysis to identify which brain regions covaried with ventral striatal activity (peak coordinates of the seed brain region: −6, 11, 0) depending on feedback valence and achievement goal orientation. Feedback valence had a significant effect on functional connectivity (corrected *p*<.048); the effect of achievement goal type and the two-way interaction effect between feedback valence and achievement goal type were not significant. This indicates that left ventral striatal activity showed greater positive interactions with the left cingulate cortex, the left precentral gyrus, the left dorsolateral prefrontal cortex (DLPFC; Figure 3A), the left inferior parietal lobe, the right postcentral gyrus, and the left superior temporal gyrus during negative feedback than during positive feedback, regardless of achievement goal orientation (Table 5). Conversely, functional interaction was no greater in any brain regions during positive feedback when compared to negative feedback. Among the brain regions that exhibited greater functional interactions with left ventral striatal activity, the pattern of BOLD signal changes in the DLPFC is presented in Figure 3B, which indicates decreased DLPFC activity regardless of feedback valence and participants' achievement goal orientation.

**Table 5:** Results of the Psychophysiological Interaction Analysis in the Multiplication Task Using the Left Ventral Striatal Activity as a Covariate.

				Talairach Coordinates			
Region	BA	Volume	Side	x	y	z	Maximum *F* value
***Negative – Positive Feedback***							
Cingulate cortex	24	1416	L	−7	−4	40	19.98
	31	496	L	−18	−31	44	15.86
Precentral gyrus	6	1200	L	−52	−7	34	19.68
Dorsolateral prefrontal cortex	9	720	L	−27	33	28	14.64
Inferior parietal lobe	40	512	L	−58	−27	26	12.00
Postcentral gyrus	3	480	R	21	−28	58	16.53
Superior temporal gyrus	22	424	L	−63	−8	3	14.75

*Note*. The cluster-wise threshold (correct *p*<.048) is determined by voxel-wise threshold (*p*<.005) and the minimum volume (53 contiguous voxels; 424 mm^3^).

#### fMRI results of the rule-finding task

**Figure 4:**
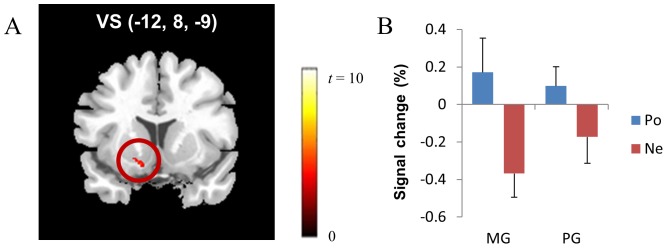
In the rule-finding task, the ventral striatum (VS) (A) showed greater neural activity during positive feedback than during negative feedback (corrected *p*<.046). BOLD signal changes in the ventral striatum were examined (B). *Note*. MG: mastery-oriented participants, PG: performance-approach-oriented participants, Po: positive feedback, Ne: negative feedback.

**Figure 5:**
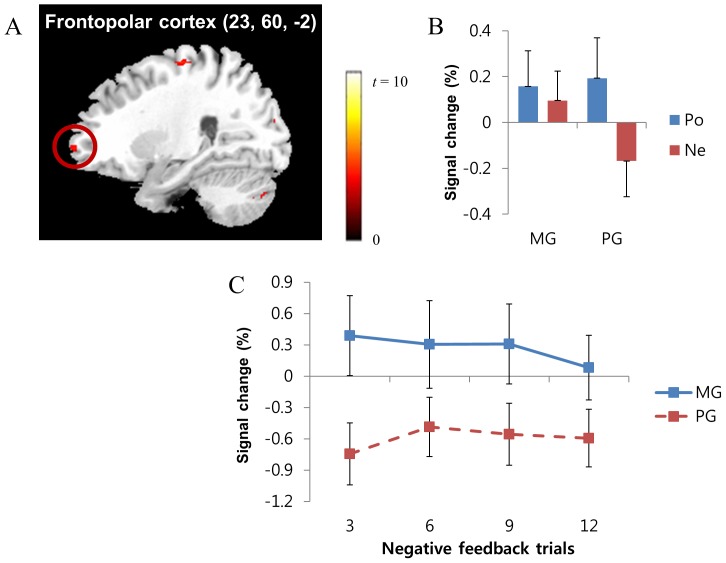
In the rule-finding task, the right frontopolar cortex (A) showed greater neural activity during positive feedback than during negative feedback (corrected *p*<.046). BOLD signal changes in the frontopolar cortex were examined (B). BOLD signal changes when receiving the first to third, fourth to sixth, seventh to ninth, and tenth to twelfth instances of negative feedback were examined separately (C). *Note*. MG: mastery-oriented participants, PG: performance-approach-oriented participants, Po: positive feedback, Ne: negative feedback.

As hypothesized, the brain regions related to reward processing (i.e., ventral striatum) were more activated during positive feedback than during negative feedback. Participants exhibited greater neural activations in the left ventral striatum (Figure 4A), the right frontopolar cortex (Figure 5A), the right thalamus, the bilateral cerebellum, the right dorsal striatum, the right occipital lobe, the right superior temporal gyrus, and the right precentral gyrus during positive feedback than during negative feedback (corrected *p*<.046; Table 6). However, no brain regions demonstrated greater neural activity during negative feedback than during positive feedback.

**Table 6:** Results of the Subtraction Analysis between Positive and Negative Feedback in the Rule-Finding Task.

				Talairach Coordinates			
Region	BA	Volume	Side	x	y	z	Maximum *t* value
***Positive – Negative Feedback***							
Ventral striatum		1928	L	−12	8	−9	5.02
Frontopolar cortex	10	1280	R	23	60	−2	4.33
Thalamus		1120	R	5	−3	8	5.46
Cerebellum		1080	R	12	−57	−30	4.70
		1032	L	−28	−77	−30	5.84
		512	R	29	−75	−30	4.12
Dorsal striatum		824	R	13	21	7	3.97
Occipital lobe	19	504	R	22	−84	29	3.78
	18	464	R	31	−82	−3	3.92
Superior temporal gyrus	22	456	R	66	−37	16	4.45
Precentral gyrus	6	456	R	25	−13	58	4.56

*Note*. The cluster-wise threshold (correct *p*<.046) is determined by voxel-wise threshold (*p*<.005) and the minimum volume (56 contiguous voxels; 448 mm^3^).

To determine the difference in brain activation patterns between positive and negative feedback for each type of achievement goal orientation, we examined BOLD signal changes in the left ventral striatum and the right frontopolar cortex as determined by the subtraction analysis between positive and negative feedback. Feedback valence had a significant effect on ventral striatal activity (*F*(1,24) = 18.39, *p*<.01), whereas the effect of achievement goal type (*F*(1,24) = .12, *p*>.05) and the two-way interaction effect between feedback valence and achievement goal type (*F*(1,24) = 2.01, *p*>.05) were not significant (Figure 4B). These neural activation patterns of the ventral striatum (i.e., increased ventral striatal activity during positive feedback vs. decreased ventral striatal activity during negative feedback) were consistent with those found in the multiplication task.

Although the achievement goal type did not have a significant effect on frontopolar cortex activity (*F*(1,24) = .38, *p*>.05), the effect of feedback valence (*F*(1,24) = 8.97, *p*<.01) and the two-way interaction effect between feedback valence and achievement goal type (*F*(1,24) = 4.52, *p*<.05) were significant (Figure 5B). That is, even though all participants showed less activation in the frontopolar cortex during negative feedback than during positive feedback, performance-approach-oriented participants had a substantial reduction in frontopolar cortex activity during negative feedback, whereas mastery-oriented participants showed only a slight reduction (but above average). To trace frontopolar cortex activation patterns during negative feedback, we examined BOLD signal changes participants received the first to third, fourth to sixth, seventh to ninth, and tenth to twelfth instances of negative feedback separately. The results revealed that mastery-oriented participants had relatively greater frontopolar cortex activity from the initial trial to the twelfth instances of negative feedback. In contrast, performance-approach-oriented participants consistently showed decreased frontopolar cortex activity regardless of when they received negative feedback (Figure 5C).

**Figure 6:**
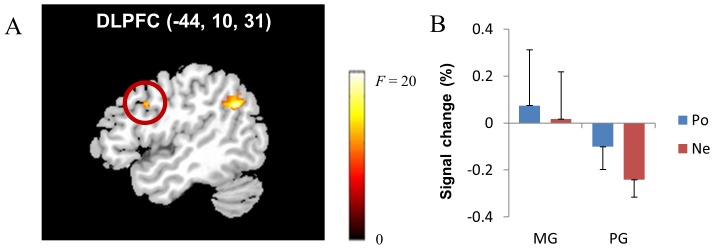
In the rule-finding task, mastery-oriented participants had a greater negative interaction between ventral striatal activity and dorsolateral prefrontal cortex (DLPFC) activity (A) during negative feedback than during positive feedback (corrected *p*<.048), whereas performance-approach-oriented participants showed a greater positive interaction. The pattern of BOLD signal changes in the DLPFC is presented (B). *Note*. MG: mastery-oriented participants, PG: performance-approach-oriented participants, Po: positive feedback, Ne: negative feedback.

We conducted PPI analyses to identify the brain regions that covaried with ventral striatal activity (peak coordinates of the seed brain region: −12, 8, −9) depending on feedback valence and achievement goal orientation. The effect of achievement goal type and the effect of feedback valence on the functional interactions between the left ventral striatum and other brain regions were not significant. However, the two-way interaction between feedback valence and achievement goal type had a significant effect on the functional interactions of the left ventral striatum with the left supramarginal gyrus, the left DLPFC (Figure 6A), and the right precuneus (corrected *p*<.048; Table 7). In particular, mastery-oriented participants produced a greater negative interaction between left ventral striatal activity and left DLPFC activity during negative feedback than during positive feedback, whereas performance-approach-oriented participants showed a greater positive interaction. The pattern of BOLD signal changes in the DLPFC is presented in Figure 6B, which indicates that mastery-oriented participants showed increased DLPFC activity while performance-approach-oriented participants showed decreased DLPFC activity regardless of feedback valence.

**Table 7:** Results of the Psychophysiological Interaction Analysis in the Rule-Finding Task Using the Left Ventral Striatal Activity as a Covariate.

				Talairach Coordinates			
Region	BA	Volume	Side	x	y	z	Maximum *F* value
***Feedback valence*** ** x** ***Achievement goal type***							
Supramarginal gyrus	40	3056	L	−39	−50	29	20.93
Dorsolateral prefrontal cortex	9	432	L	−44	10	31	14.34
Precuneus	7	424	R	4	−70	37	17.18

*Note*. The cluster-wise threshold (correct *p*<.048) is determined by voxel-wise threshold (*p*<.005) and the minimum volume (53 contiguous voxels; 424 mm^3^).

## Discussion

The results of the behavioral experiment clearly indicated that mastery goals had a positive relation with challenge seeking both before and after experiencing difficulty in task performance. This is consistent with achievement goal theory, which postulates that mastery-oriented individuals tend to seek challenge regardless of the presence of difficulty or obstacles [3], [4]. Although previous achievement goal research has suggested that performance-oriented individuals are more likely to avoid challenge due to fear of failure [11], the relation between performance-approach goals and challenge seeking remains unclear [7], [9]. The results of our behavioral experiment revealed that performance-approach goals were not related to pre-task challenge seeking but had a negative relation with post-task challenge seeking. This suggests that performance-approach-oriented individuals are more likely to avoid challenge in the presence of difficulty.

Achievement goal theorists have used the confounding effect of confidence to explain the inconsistency between pre- and post-task challenge seeking [4], [11]. When performance-approach-oriented individuals experience failure or difficulty, they tend to become less confident in their ability, which in turn can reduce their tendency to seek challenge. However, if they do not encounter difficulty, they are likely to remain confident in their ability, and thus they may not avoid challenge. The results of our behavioral experiment provide support for this hypothesis; math self-efficacy had a positive relation with challenge seeking before difficulty was encountered during task performance, whereas it had no significant relation with challenge seeking after the experience of difficulty during task performance. This suggests that performance-approach-oriented individuals lose their confidence in the face of difficulty and tend to avoid challenge.

To understand these differences between mastery-oriented and performance-approach-oriented individuals in challenge-seeking behavior in the face of difficulty, we need to understand how these two groups perceive negative feedback during task performance. In terms of neural activity, the ventral striatum played a key role during feedback processing, in both the multiplication and rule-finding tasks. The results indicated that participants generated more ventral striatal activity during positive feedback than during negative feedback regardless of achievement goal orientation and task type. This is consistent with previous findings related to reward and feedback processing. The ventral striatum is generally activated by valence of rewards [31]–[34] and also recruited when feedback clearly indicates whether the performance is good or bad [13], [35], [36].

However, the strength of the ventral striatum–DLPFC coupling during negative feedback varied significantly depending on achievement goal orientation and task type. In the multiplication task, we did not find any differences between mastery-oriented and performance-approach-oriented participants in terms of the strength of the ventral striatum–DLPFC coupling and the magnitude of DLPFC activity. Although both mastery-oriented and performance-approach-oriented participants showed greater functional connectivity between ventral striatal and DLPFC activity during negative feedback than during positive feedback, they showed decreased DLPFC activity during both positive and negative feedback regardless of achievement goal orientation. This indicates that both mastery-oriented and performance-approach-oriented individuals do not recruit this control mechanism during feedback processing.

The rule-finding task, by contrast, produced a negative coupling between the ventral striatum and the DLPFC during negative feedback for mastery-oriented participants, and a positive coupling for performance-approach-oriented participants. Because of this connectivity difference, mastery-oriented participants showed relatively increased DLPFC activity during negative feedback, whereas performance-approach-oriented participants showed relatively decreased DLPFC activity. The ventral striatum–DLPFC interaction might be a key neural mechanism in understanding negative feedback processing in the rule-finding task. Since the DLPFC is known to be responsible for the control of cognition, valuation, and behavior [37], [38], particularly during negative feedback processing [18], [19], these findings suggest that mastery-oriented individuals are more likely to recruit control processes when confronted with negative feedback in order to accomplish a challenging task than are performance-approach-oriented individuals. In contrast, performance-approach-oriented individuals are less likely to engage in control processes when they receive negative feedback.

Participants who differed in achievement goal orientation also showed considerable differences in the frontopolar cortex activity during negative feedback in the rule-finding task. Mastery-oriented participants did not show substantial reduction in the frontopolar cortex activity during negative feedback when compared to positive feedback. Particularly, in the initial stages of the rule-finding task, they demonstrated relatively high levels of frontopolar cortex activity even during negative feedback. In contrast, performance-approach-oriented participants showed a significant decrease in frontopolar cortex activity during negative feedback but an increase during positive feedback. They also consistently exhibited low levels of frontopolar cortex activity whenever they received negative feedback at any time during the rule-finding task.

The frontopolar cortex is known to be associated with higher-order cognitive functions [20], [39]–[41]. It has been suggested that the frontopolar cortex works as a supervisory system [40], allocates sub-processes [20], [42], or represents multiple cognitive outcomes [21], [39]. Despite diverse opinions on the specific role of the frontopolar cortex, it is generally agreed that the frontopolar cortex is recruited when individuals attempt to retain a long-term goal during the execution of subordinate functions. In the rule-finding task of this study, the participants may have used task-relevant information from feedback to determine whether to maintain their current guess regarding the unknown rule or shift to an alternative. Therefore, performance-approach-oriented individuals are less likely to focus on the task-relevant information of the negative feedback, whereas mastery-oriented individuals are more likely to focus on the constructive elements of the negative feedback in order to achieve their task goal, perceiving negative feedback as informative for subsequent performance even though this tendency can be weakened by repeated failure.

## Conclusions

The ultimate goal of this study was to examine the relations of achievement goals to challenge seeking and brain responses during both positive and negative feedback processing. The results indicate that performance-approach-oriented participants had a substantial reduction in challenge-seeking behavior and satisfaction with negative feedback after experiencing difficulty during task performance, while mastery-oriented participants demonstrated little or no reduction. These results suggest that individuals with different achievement goal types use fundamentally different mechanisms to process negative feedback and seek challenge. That is, mastery-oriented individuals, who emphasize learning and mastering something for its own sake, are more likely to focus on the informative nature of negative feedback and take a proactive stance with respect to difficulty. In contrast, performance-approach-oriented individuals, who look to outperform others and thus demonstrate their ability, are more likely to focus on the confirmative nature of negative feedback and take a reluctant stance towards difficulty.

There are three possible limitations in this study. First, maladaptive types of goals (e.g., performance-approach goals) were not considered in this study. We believe that the debate within the achievement goal literature is between mastery versus performance-approach goals in terms of motivation and performance as there is no controversy in the negative consequences of the maladaptive types of goals. Therefore, we only focused on the comparison of the adaptive types of goals (i.e., mastery and performance-approach goals) in this study. Second possible limitation is a possibility of multiple goal adoption. In the fMRI experiment, we compared the mastery-oriented and performance-approach-oriented groups. However, some researchers have suggested that there could be individuals with high performance-approach goals together with high mastery goals [43]. In this regard, it is also necessary to examine the independent achievement goal effects on neural activity during feedback processing in future studies. Third, due to the fixed two-second interval between the task and feedback stimuli, neural activity during feedback processing could not be separated out from neural activity during task performance. Despite this possible confounding effect, we did not use a randomized inter-stimulus interval in order to prevent participants from carrying out unpredictable mental processes to varying degree. Even though we used a fixed two-second inter-stimulus interval, we were able to minimize the confounding effect of the two tasks (i.e., multiplication task vs. rule-finding task) on neural activity during feedback processing by only comparing neural activity across conditions when participants were performing the same task.

Even considering these limitations, there is an important implication from the findings of the present study. On the path to learning and expertise, it is inevitable that learners will experience failure and difficulty. Thus, to attain learning goals and develop expertise, learners need to be willing to confront their failure and overcome difficulty [44]. The process of actively overcoming difficulty can provide an opportunity to practice skills and hone abilities. However, individuals who avoid challenge and difficulty may lose this opportunity. Therefore, we can conclude that the reluctant stance associated with performance-approach goals is maladaptive in challenging situations, whereas the proactive stance associated with mastery goals is more adaptive.
